# An Odd Crisis: Covid‐19 and UK Food Prices

**DOI:** 10.1111/1746-692X.12291

**Published:** 2021-02-07

**Authors:** Han Lin, Tim Lloyd, Steve McCorriston

**Affiliations:** ^1^ University of Exeter UK; ^2^ Bournemouth University UK; ^3^ University of Exeter UK

## Abstract

The Covid‐19 pandemic will have impacts that will vary across countries and commodity sectors, reflecting factors such as the importance of trade, differences in the functioning of supply chains and the market which producers and farmers supply. Some of these effects will be relatively short‐lived; others will be longer‐lasting. In this context, we set out the channels through which food prices will be affected by the Covid‐19 pandemic, emphasising the short‐ and longer‐term nature of the main effects. We focus on the UK but the insights extend to other (importing) countries. Drawing on a recent econometric model of UK retail food prices that accounts for both domestic and international factors, we show that the key drivers have potentially off‐setting effects, suggesting that the Covid‐19 shock to the food sector is likely to be different from previous shocks, particularly the commodity price crises of 2007–2008 and 2011. In many European countries, the Covid‐19 pandemic may manifest itself as something of an ‘odd crisis’, in which lower world and farm‐gate prices co‐exist with higher domestic retail prices. These off‐setting factors will frame policy responses targeted at different stages of the food chain across countries.

## Abstract (fr)

Les incidences de la pandémie de Covid‐19 varieront selon les pays et les secteurs de produits, reflétant des facteur stels que l'importance des échanges, les différences dans le fonctionnement des chaînes d'approvisionnement et le marché que les producteurs et les agriculteurs approvisionnent. Certains de ces effets seront de relativement courte durée; d'autres plus persistants. Dans ce contexte, nous avons défini les canaux de transmission des effets de la pandémie de Covid‐19 sur les prix des denrées alimentaires, en insistant sur la nature à court et à long terme de ces principaux effets. Nous nous concentrons sur le Royaume‐Uni, mais les conclusions s’étendent à d'autres pays (importateurs). En nous inspirant d'un récent modèl eéconométrique des prix alimentaires de détail au Royaume‐Uni, qui tient compte des facteurs nationaux et internationaux, nous montrons que les principaux facteurs ont des effets potentiellement compensateurs, ce qui suggère que le choc de la pandémie de Covid‐19 sur le secteura limentaire pourrait être différent des chocs antérieurs, en particulier les crises des prix des produits de base de 2007–2008 et 2011. Dans de nombreux pays européens, la pandémiede Covid‐19 peut se manifeste rcommeune sorte de « crise étrange », dans laquelle la baisse des prix mondiaux et des prix au niveau de la ferme coexiste avec des prix intérieurs de détail plus élevés. Ces facteurs compensatoires en cadreront les réponses ciblées entermes d'action publique à différents stades de la chaîne alimentaire à travers les pays.

## Abstract (de)

Die Auswirkungen der Covid‐19‐Pandemie werden von Land zu Land und von Sektor zu Sektor unterschiedlich sein und spiegeln Faktoren wie die Bedeutung des Handels und Unterschiede in der Funktionsweise der Lieferketten sowie des Marktes, den Produzierende und Landwirtinnen und Landwirte beliefern, wider. Einige dieser Auswirkungen werden relativ kurzlebig sein, andere werden länger andauern. In diesem Zusammenhang zeigen wir die Kanäle auf, über welche die Lebensmittelpreise durch die Covid‐19‐Pandemie beeinflusst werden. Dabei heben wir die kurzfristigen und längerfristigen Hauptauswirkungen hervor. Unser Fokus liegt auf dem Vereinigten Königreich; die Erkenntnisse erstrecken sich jedoch auch auf andere (importierende) Länder. Auf der Grundlage eines aktuellen ökonometrischen Modells zu den Lebensmittelpreisen im britischen Einzelhandel, das sowohl inländische als auch internationale Faktoren berücksichtigt, zeigen wir, dass die wichtigsten Treiber potenziell gegenläufige Effekte haben. Dies deutet darauf hin, dass sich der Covid‐19‐Schock im Lebensmittelsektor wahrscheinlich von früheren Schocks unterscheidet, insbesondere von den Produktpreiskrisen der Jahre 2007–2008 und 2011. In vielen europäischen Ländern kann sich die Covid‐19‐Pandemie als eine Art „seltsame Krise” zeigen, in der niedrigere Welt‐ und Erzeugerpreise mit höheren inländischen Einzelhandelspreisen koexistieren. Diese gegenläufigen Faktoren werden den Rahmen für politische Maßnahmen bilden, die auf die verschiedenen Stufen der Lebensmittelkette in den einzelnen Ländern abzielen.

As the Covid‐19 pandemic unfolds, what is becoming apparent is that we are experiencing a shock like no other in modern times; global in scale and unprecedented in severity. What began as an acute public health crisis has morphed into a collection of crises engulfing health, financial markets and supply chains in almost all countries and with economic repercussions on a scale not seen since the Great Depression. Despite allegedly originating in a food market, Covid‐19 is not a food crisis *per se* but a disease pandemic with significant consequences for food chains and, as such, its effects are unlike those that characterise typical food crises.

“Contrairement aux crises précédentes, les réponses du gouvernement et des parties prenantes se placeront dans un contexte où la baisse des prix agricoles mondiaux et intérieurs coexistera avec des prix alimentaires au détail plus élevés.”

**Figure:**
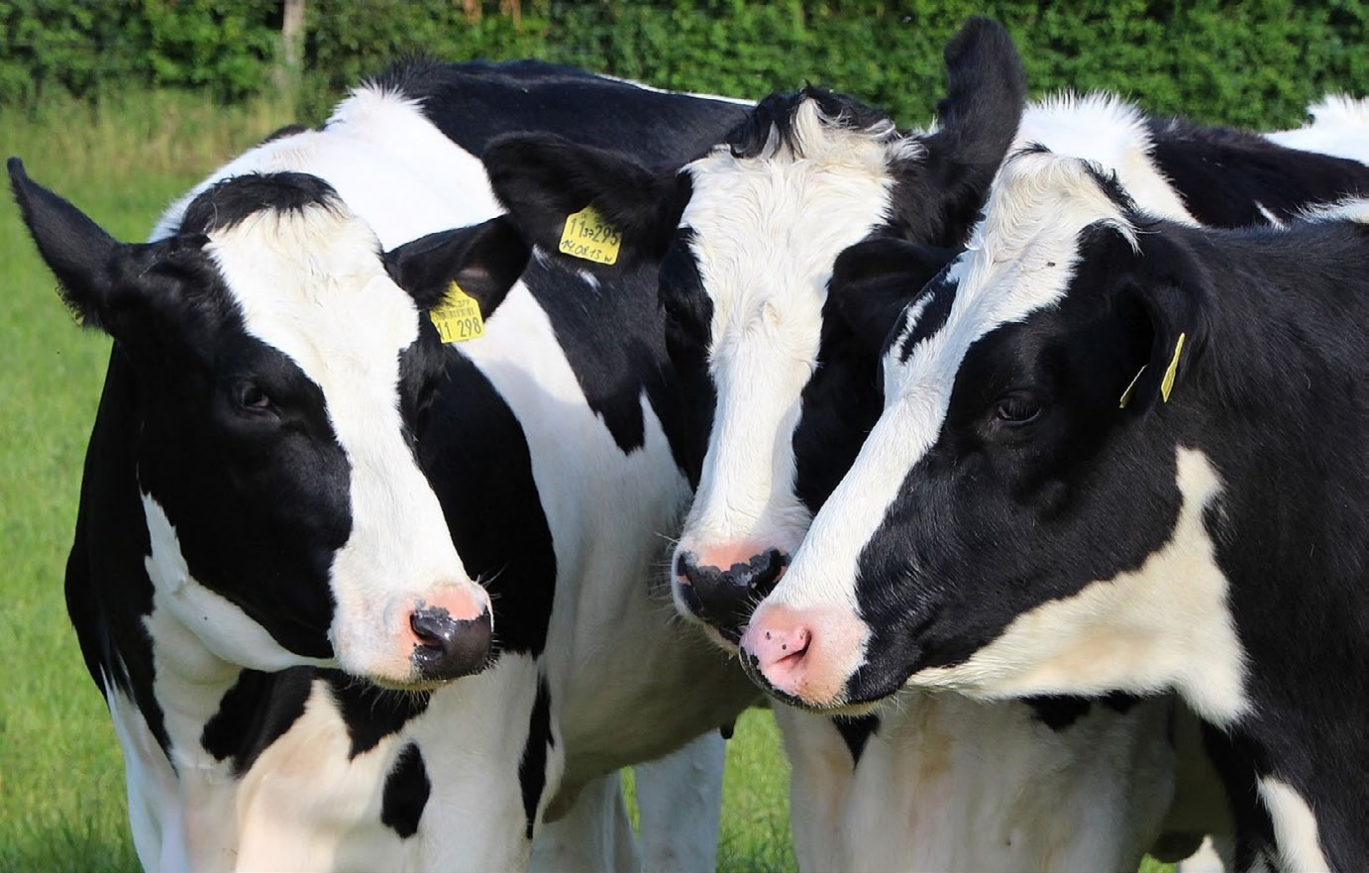
Dairy farmers have been among the hardest hit by falling demand.

In this article, we address how the emerging impacts of Covid‐19 may affect the consumer price of food. While our attention focusses on the UK, important insights from this research extend to other developed countries reliant on food imports. We argue that Covid‐19 is unlikely to trigger a spike of food inflation, the signature of all previous crises, largely because it will induce a number of off‐setting effects. Moreover, we are likely to observe consumer and producer prices moving in opposite directions, again in contrast to previous crises. While some of the factors will impart short‐run impacts on food prices, others will be more long‐lasting, framing policy responses towards agriculture and the food sector alongside other substantive issues including climate change and, more specifically to our focus on the UK, Brexit. To address the extent of these potentially off‐setting effects of the Covid‐19 pandemic, we draw on an extension of Davidson *et al*.'s (2016) econometric model of UK retail food inflation that accounts for both international and domestically‐sourced shocks. We do not use the model to forecast food prices but rather to illustrate the potentially offsetting effects of Covid‐19. While the model has forecasting capability, we refrain from using it for this purpose, because of the uncertainty regarding the timing and severity of Covid‐19 across the range of drivers in the model, and because of another great source of uncertainty, namely Brexit, that will also affect food prices.

## ‘This time is different’

Shocks to agricultural and food sectors are common but they are typically confined to specific commodity sectors or country contexts, such as extreme weather events or food scares. Global commodity‐related shocks affecting all countries and covering a wide array of commodity sectors are far less common. When they do occur, they typically give rise to spikes in commodity prices, such as those in 2007–2008 and 2011, and bursts of food price inflation (UK food inflation peaked at 13 per cent in 2008). It is noteworthy that those commodity price spikes were associated with a number of inter‐related factors: poor harvests in key exporting countries; low and uncertain stocks of commodities worldwide; high oil prices; the diversion of agricultural land into the production of biofuels; strong demand in rapidly growing Asian economies; and the financialisation of commodity trading. All exerted upward pressure on the price of agricultural commodities and food. They also led – from a global perspective at least – to trade policy responses which served to accentuate the problem.

The Covid‐19 context has few of these concerns: oil prices have fallen to historically low levels as the global economy contracts; forecasts confirm good harvests for this year; stocks are high; concerns over the impact of biofuel demand have receded; and the use of export restrictions has been limited, so far. Taken together, the Covid‐19 impact on food and agricultural markets is occurring in the context of relatively low prices and sufficient stocks. While response to weak prices may reduce supplies in the longer term, global availability of commodities is not the main issue currently.

## Covid‐19 impacts

So, what are the forces at play emerging from the Covid‐19 pandemic? Here we highlight a combination of macroeconomic and food‐chain specific factors that have potentially offsetting effects, making commodity price spikes and food inflation unlikely consequences of the Covid‐19 crisis. Furthermore, these factors are likely to drive farm and food prices in opposite directions. Unlike previous crises, policy and stakeholder responses will be framed in a setting where lower world and domestic agricultural prices co‐exist with higher retail food prices.

### Demand‐side factors

The International Monetary Fund has recently forecast significant reductions in economic growth in 2020 and reduced earlier growth forecasts for 2021. IMF (2020) forecasts a reduction of c.5 per cent in the world economy in 2020, 8 per cent in the US and 10 per cent in the UK and euro area. Recent data from the OECD are more alarming on the immediate macroeconomic consequences: GDP in the UK is forecast to decline by 11.5 per cent between 2020 and 2021; and by 14 per cent if there is a second wave of infection (OECD, 2020a). The expectation is of a deep and prolonged recession as countries transition at different speeds from Global Lockdown amidst the unemployment, lower income and debt that will be extant long after the medical emergency recedes.

“Im Gegensatz zu früheren Krisen werden die Reaktionen der Politik und der Interessengruppen in einem Szenario erfolgen, in dem niedrigere weltweite und inländische Agrarpreise mit höheren Lebensmittelpreisen im Einzelhandel koexistieren.”

While the demand for food and drink is more resilient to changes in consumer income than other categories of expenditure, such as clothing and automobiles, the reliance on food banks by an increasing portion of the population in seemingly affluent societies reminds us that food poverty is as much a part of the new normal as social distancing. Aside from the acute impact on vulnerable groups, the demand‐side effect is more nuanced as the main mechanism will relate to *where* food is consumed. In many developed countries, food‐away‐from‐home accounts for a large proportion of food consumption. In the US, it is 55 per cent of total food expenditure; in the UK, it is around 30 per cent. While the decrease in food consumed outside the home will increase food purchased at supermarkets, consumption in these two segments of the retail food market are not perfect substitutes. When incomes and employment do recover, attitudes to eating out and long‐lasting restrictions on restaurant capacity may well mean that a decline in the demand for food‐away‐from‐home will be with us for some time to come. As the macroeconomic projections noted above imply, the impact on food supply chains, and agriculture in particular, will be more long‐lasting due the relative importance of the food‐away‐from‐home channel.

The closure of food service outlets has had acute short‐term impacts throughout the food chain. Falling demand from coffee shops and caterers has resulted in depressed farm‐gate prices and to media reports of dairy farmers dumping milk; milk prices have declined by around 25 per cent, making production unviable for a large number of farm businesses (*Financial Times*, 20 April 2020). While potentially transitory, this is not an isolated incident with the experience being common across other countries and other sectors; beef, cheese and potato sectors being particularly vulnerable to the sharp demise in food hospitality outlets and food service more generally (*Financial Times*, 1 May 2020). Food suppliers have also been affected. Retail supermarkets have well defined, vertically‐coordinated supply chains that make it difficult to shift farm output from one supply channel to another. Hence, demand‐side impacts not only affect farmers but also a much more economically significant food service sector that is exposed to the potentially long‐lasting decline in the food‐away‐from‐home channel.

### Supply‐side factors

Given the apparent ample supplies of commodities, supply‐side factors are more likely to manifest themselves as problems involving higher health and safety costs, logistics and labour availability. While some adjustments in the food chain were short‐lived (e.g. expanded home delivery to self‐isolating groups and absenteeism), others seem set to be more persistent, being associated with a much broader set of longer‐term operational changes that will increase the cost of growing, processing and distributing food to the consumer. For example, restrictions on the movement of goods and labour, essential for harvesting and processing, and heightened border checks will increase costs. In the UK, in particular, the lack of adequately trained and available domestic labour for harvesting and processing fresh foods will either lead to substitution of capital or lost output, both of which increase prices in the short/medium term. While these costs seem set to increase the price of food at retail, it is important to recognise that changes in energy costs may partially offset this inflationary pressure if the slump in world oil prices is fully transmitted into energy‐intensive food chains.

### Trade and exchange rates

The World Trade Organisation forecasts a significant reduction in international trade as a consequence of the pandemic (WTO, 2020). While much of the discussion has focused on the vulnerability of supply chains in manufacturing, trade in agricultural and food products will also be affected. IFPRI estimates that world agricultural trade will decline by around 25 per cent (Laborde *et al*., 2020).

“Unlike previous crises, policy and stakeholder responses will be framed in a setting where lower world and domestic agricultural prices co‐exist with higher retail food prices.”

In the face of weak prices globally for agricultural commodities, what will matter for small open economies like the UK is the price at the border, not the world price *per se*. There are two factors to appreciate here. One relates to logistics. Restrictions on air cargo and shipping have affected the movement of goods; air cargo rates – important for high value foods such as fruit and vegetables – increased by around 60 per cent in the tempest of the pandemic (OECD, 2020b) but, longer term, the depressed price of oil is likely to play an off‐setting role.

Second, since most agricultural products are priced in dollars, exchange rates also matter to the domestic price of commodities traded on world markets. Exchange rate changes can offset or reinforce commodity price changes for all food importers, but are particularly relevant to the UK where food self‐sufficiency is around 50 per cent. Davidson *et al*. (2016) report that the transmission elasticity for exchange rates to UK retail prices is greater than that arising from world commodity prices, owing to the importance of non‐agricultural components in retail food. As we confirm below, the effect of international drivers will be key to addressing the overall impact of Covid‐19 on UK food prices.

## UK food price impacts

One of the key messages from this article is that, unlike previous crises, the effects of Covid‐19 on food prices are potentially offsetting, with the result that spikes in food inflation that characterised virtually all previous crises seem unlikely. To illustrate, we use the parameters of a recently developed econometric model, details of which are provided in Box 1. We use the model not to simulate various Covid‐19 scenarios but to highlight our central point. As such, our ambition is rather modest, but for good reason: there is simply too much uncertainty regarding the length and severity of the pandemic to offer reliable forecasts at present, all the more so given the on‐going negotiations over a Brexit trade deal with the EU. We can nevertheless gain some insight into the prospects for retail food prices in the UK by applying the *direction of change* (about which there is more confidence) to the model's parameters.

**Econometric model**

The model reported here is an extension of Davidson *et al*.'s (2016) time series model of the drivers of food inflation in the UK. It has two key components: a relationship that links *domestic drivers of food prices* (labour costs, import prices, domestic agricultural prices and non‐labour manufacturing costs) and a second relationship linking *import prices of food and agricultural commodities* with domestic agricultural price and exchange rates. These two relationships tie domestic and international drivers of retail food prices together and allow for the inter‐relationships between all variables in the form of a co‐integrated vector autoregressive model, which is estimated using monthly data over the 1997(1) to 2017(6) period.

An innovative feature of the model is that it does not employ the more generic world commodity price indices to measure raw material costs, but the actual prices of UK imports obtained directly from HMRC trade data. This import price therefore accommodates two important aspects relevant to Covid‐19. Prices of agricultural and processed food products will not only reflect sourcing but, because they are prices at the UK border, they will also reflect transportation costs. In addition, the exchange rate used in the modelling is an effective exchange rate of pound sterling against the Euro and the US dollar with the shares weighted by the importance of EU countries (that account for a large proportion of UK food and agricultural imports) as well as non‐EU countries.

Table 1 sets out the direction of change to the main drivers that Covid‐19 may be expected to induce. The first column highlights international factors, the second column identifies those that are domestic in nature and the last column focuses on the principal effect in each category. Those that are likely to put upward pressure on food prices include exchange rates, manufacturing costs and labour costs; those that are likely to depress food prices include domestic and import agricultural prices.Overall, three factors are expected to have inflationary effects and two are deflationary, although given that non‐labour manufacturing costs include the cost of energy, its net effect is less clear. To indicate the relative magnitudes of implications of the model's parameters,we consider an arbitrary 10 per cent change (positive for those drivers likely to increase, negative for those likely to fall). While the size of the shock is arbitrary, the results can be readily interpreted for any percentage shock to each driver that readers choose to impute. Summing individual effects gives the net effect on food prices of a set of conjectured shocks.

**Table 1:** The anticipated effect of Covid‐19 food price drivers in the UK

	International	Domestic	Main Effects
Labour Costs	‐	Increase	‐
Other Manufacturing Costs	‐	Increase	‐
Domestic Agricultural Prices	‐	Decrease	Decrease
Import Prices	Decrease	‐	‐
Exchange Rate	Increase	‐	Increase

**Figure 1:**
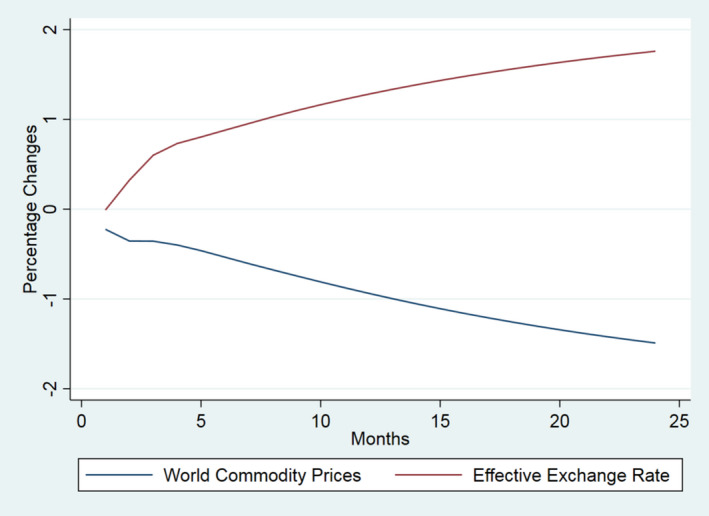
The dynamic effect of 10 per cent shocks to main drivers of UK food prices (per cent): Key international drivers

**Figure 2:**
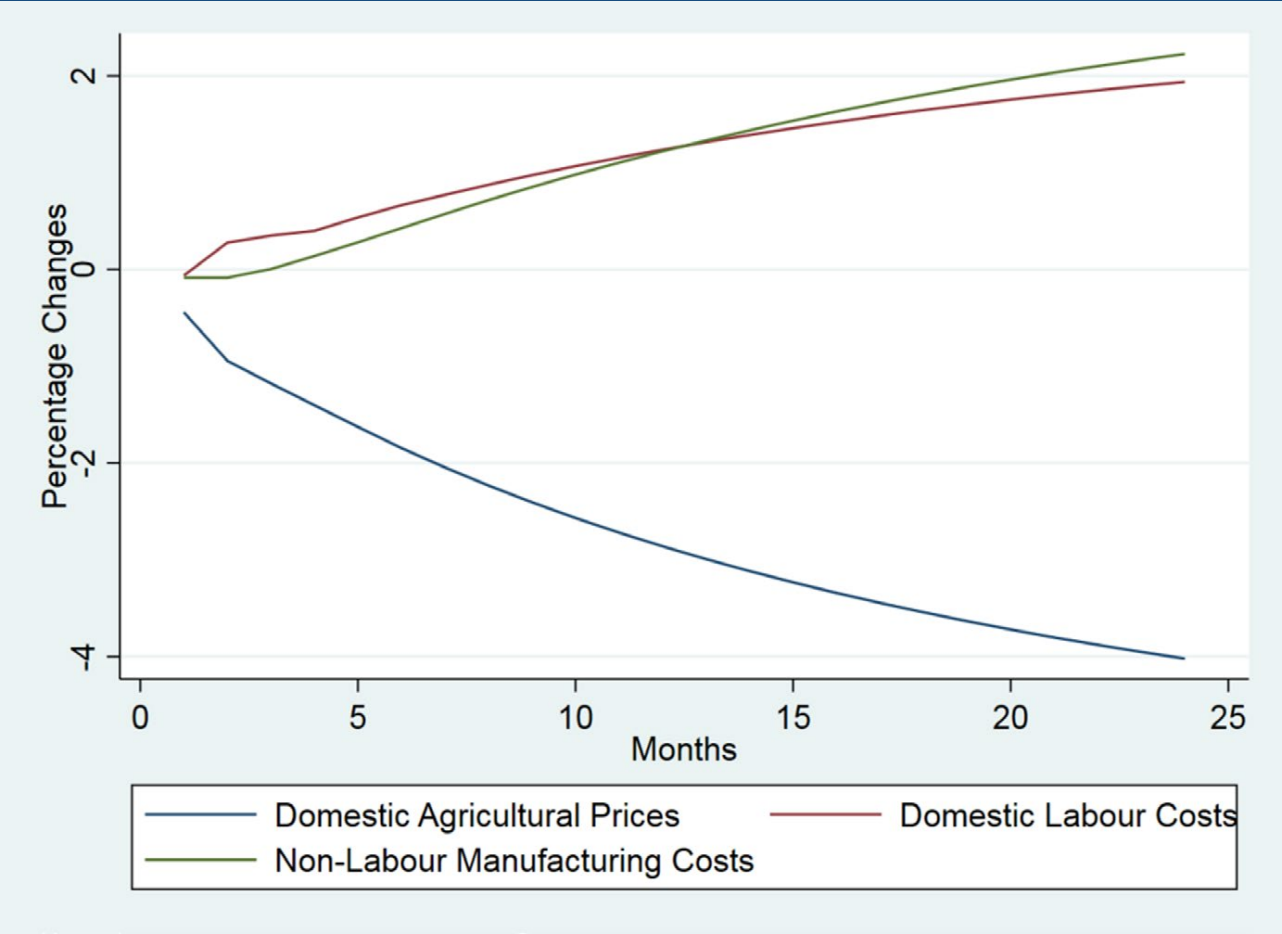
The dynamic effect of 10 per cent shocks to main drivers of UK food prices (per cent): Key domestic drivers

**Figure 3:**
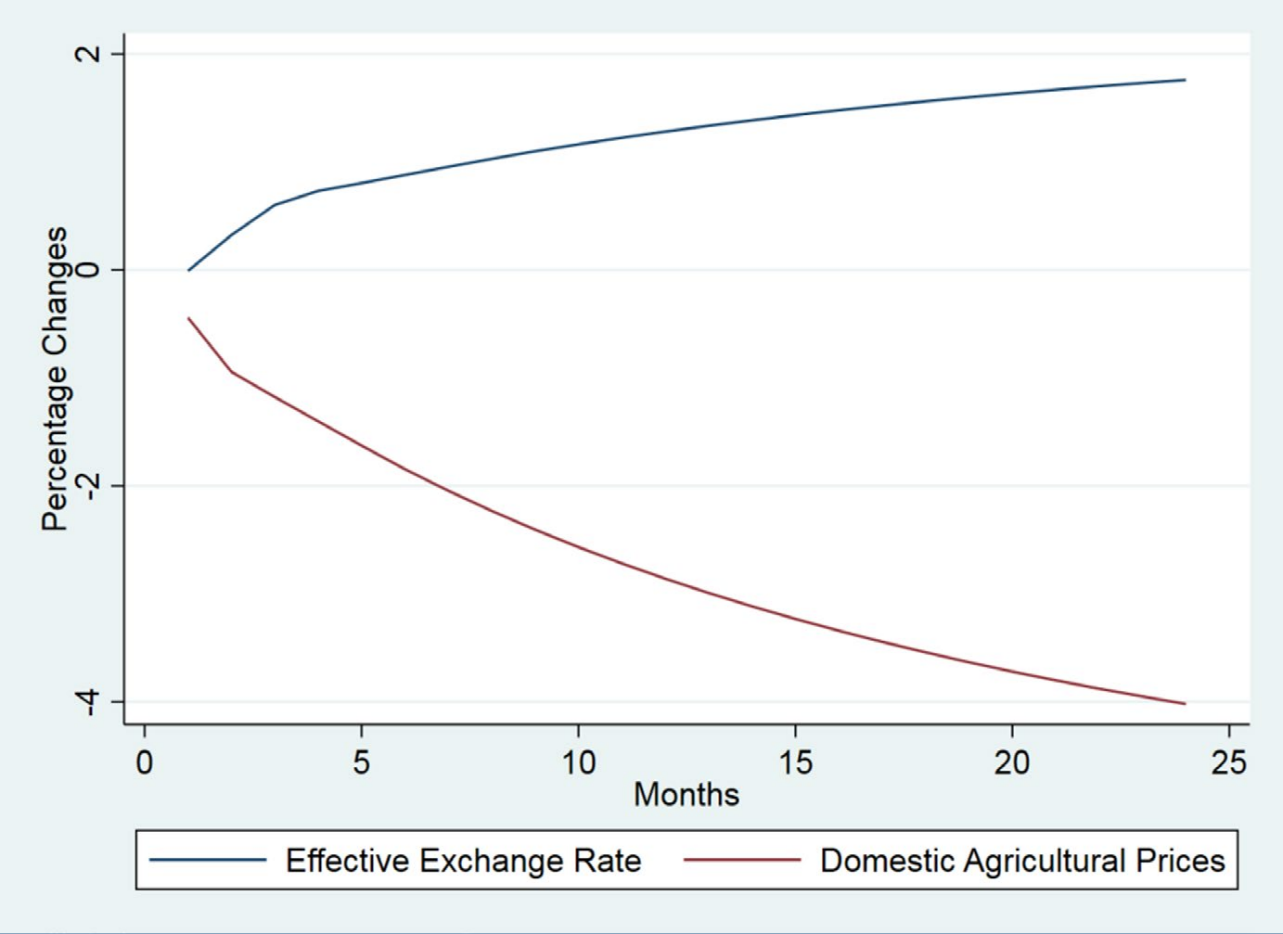
The dynamic effect of 10 per cent shocks to main drivers of UK food prices (per cent): Exchange rates and domestic agricultural prices

For ease of interpretation, and to illustrate the offsetting nature of the Covid‐19 effects, the results are presented in the three groups of Table 1. In Figure 1 we show the effect of 10 per cent shocks to each of the international factors (i.e. the UK import price for food and agricultural products and the effective exchange rate). In Figure 2, we shock only domestic factors (i.e. domestic agricultural prices, UK labour costs and non‐labour manufacturing costs). In Figure 3, we compare the most dominant international shock (the effective exchange rate) with the most dominant domestic driver (domestic agricultural prices).

### Results

All shocks result in less than perfect transmission (i.e. effects are less than proportional), with a majority (>50 per cent) of adjustment occurring within 6 and 11 months depending on the source of the shock, implying that retail prices *per se* respond slowly, with delayed yet persistent effects. Moreover, they show that Covid‐19 may be expected to impart effects that are offsetting with respect to both the domestic and international drivers. The magnitude of the transmission of shocks to retail prices varies across the drivers. As declared above, our aim here is not to forecast the actual effect of Covid‐19 but simply to show the potentially off‐setting nature of the drivers involved; the actual outcome will depend on the magnitude of the change in each driver and how long each lasts.

The results highlight that, in contrast to previous and more typical shocks to food markets in the recent past, Covid‐19 is potentially unusual due to the off‐setting effects it is likely to trigger. So, while there is too much uncertainty to predict the actual effect of Covid‐19, there is good reason to suggest that the largest shock to the world economy since the Great Depression may potentially have limited effects on the consumer price of food, largely due to the off‐setting direction of changes in the drivers of food inflation. Admittedly, some drivers may change direction or be exacerbated due to Brexit, as noted below.

## Policy implications

The Covid‐19 shock to the food sector in the UK and beyond has already generated a wide range of responses from policymakers. Most notably, the furloughing of labour has maintained household incomes, easing the immediate burden for small businesses of rent and other financial commitments, while support for the farm sector has also been made available (for details see AgraFocus, May 2020). Many of these measures represent responses to the immediate impact of Covid‐19 on the food chain. But what additional policy insights arise from the modelling framework presented above? We highlight three issues of note.

1. *Dynamics*. While many supply‐side drivers (e.g. plant and restaurant closures, logistics, home delivery and social distancing in the food processing and retailing sectors) will be acute only during the Lockdown phase, some responses such as the greater reliance on automation will have more persistent effects on the costs and structure of businesses in the food chain. Demand‐side factors are also likely to impose a longer‐term impact on the food sector. Recent OECD forecasts on the anticipated macroeconomic impact of Covid‐19 (through unemployment, debt and disposable income effects) suggests that slow recovery of demand in the food service sector will result in sustained pressure on the upstream sectors of the food chain, given the importance of food‐away-from‐home in household food expenditure.
2. *Sectoral Differences*. If the effect on consumer prices turns out to be relatively modest, this does not mean that there will not have been significant adjustment in the food sector. The financial stress inflicted by the pandemic itself and the measures to alleviate its spread will continue to squeeze the notoriously thin profit margins in the food sector. The SMEs that proliferate in the sector as a whole, but particularly in food service, are vulnerable to weak demand and it is here where structural change is likely to be most acute. With supermarket prices rising due to exchange rate effects and higher logistical costs, demand for farm products will weaken, compounding labour shortages and declining prices due to the demand‐side influence of the food service and hospitality sector.
3. *Policy Responses*. While there is little indication of immediate concern over global supplies, if domestic farm prices weaken as a consequence of the demand‐side impacts in domestic markets, the pressure for government intervention to aid agriculture or support farm prices will intensify. More broadly, the concern is that Covid‐19 will be used to demonstrate the sector's vulnerability to external shocks, and invoke a more nationalistic policy stance.

**Figure:**
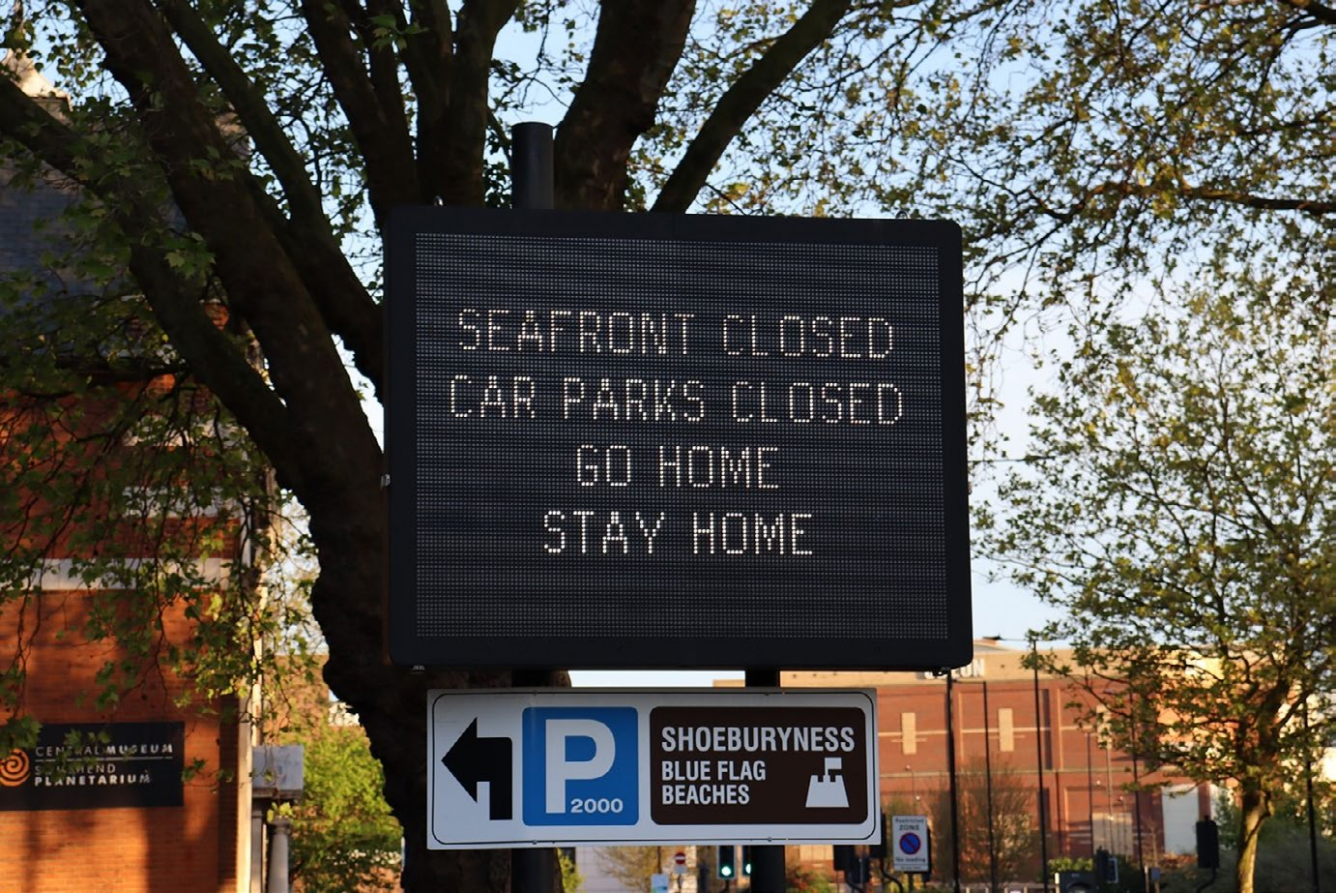
The hospitality sector has been worse hit and will remain restricted for longer.

## Covid‐19 versus Brexit

Our discussion so far has set aside any impact on food prices that may arise from the UK's departure from the European Union; but one way or another, Brexit has the potential to have a significant effect on food prices. There are two aspects that are relevant here. The first is the most obvious: in the absence of a trade deal, barriers to trade on agricultural and food imports from the EU will rise, feeding through into higher retail prices, potentially offsetting weaker commodity prices. Some assessment of alternative scenarios across different farm sectors is available from the FAPRI‐UK project (Davis *et al*., 2017). But this is not the only Brexit effect that would arise in our framework. As Breinlich *et al*. (2019) have recently noted, the UK's decision to leave the EU has been accompanied by a significant depreciation of Sterling, which is itself a potent source of inflationary pressure compounding any Covid‐related depreciation. The insight here is that UK food prices will not be immune to the macroeconomic consequences that arise from Brexit. In short, the combination of Covid‐19 and Brexit provide a cocktail of challenges to the UK food sector.

## Off‐setting factors

The Covid‐19 pandemic will certainly affect food sectors across the world but, in many ways, it creates an unusual crisis. Given that it is not due to a global shortage of food and agricultural products, changes in food prices will be driven by demand‐side effects and higher health and logistical costs. While there is no immediate pressure on world prices, that does not mean that import prices cannot rise, since trade policy may also be influential, particularly in the UK in respect of Brexit. Similarly, the functioning of domestic food supply chains and the extent to which the restaurant and hospitality sector recover will also be pivotal. The main insights from the econometric results is that the impact of Covid‐19 on retail food prices in the UK will involve a range of factors both domestic and international that will likely be largely off‐setting. These off‐setting influences may mask some of the more acute pressures that arise at different stages of the food chain; rising retail food prices being perfectly consistent with falling farm‐gate prices in the face of higher marketing costs. Given the sensitivity over food prices generally, the regressive nature of food price inflation for food consumers and the issues that will arise relating to the dependence of consumers on food imported from abroad, Covid‐19 will certainly bring into sharper focus the need for food supply chains to be made more resilient to shocks originating from both domestic and external drivers of food prices.

**Figure:**
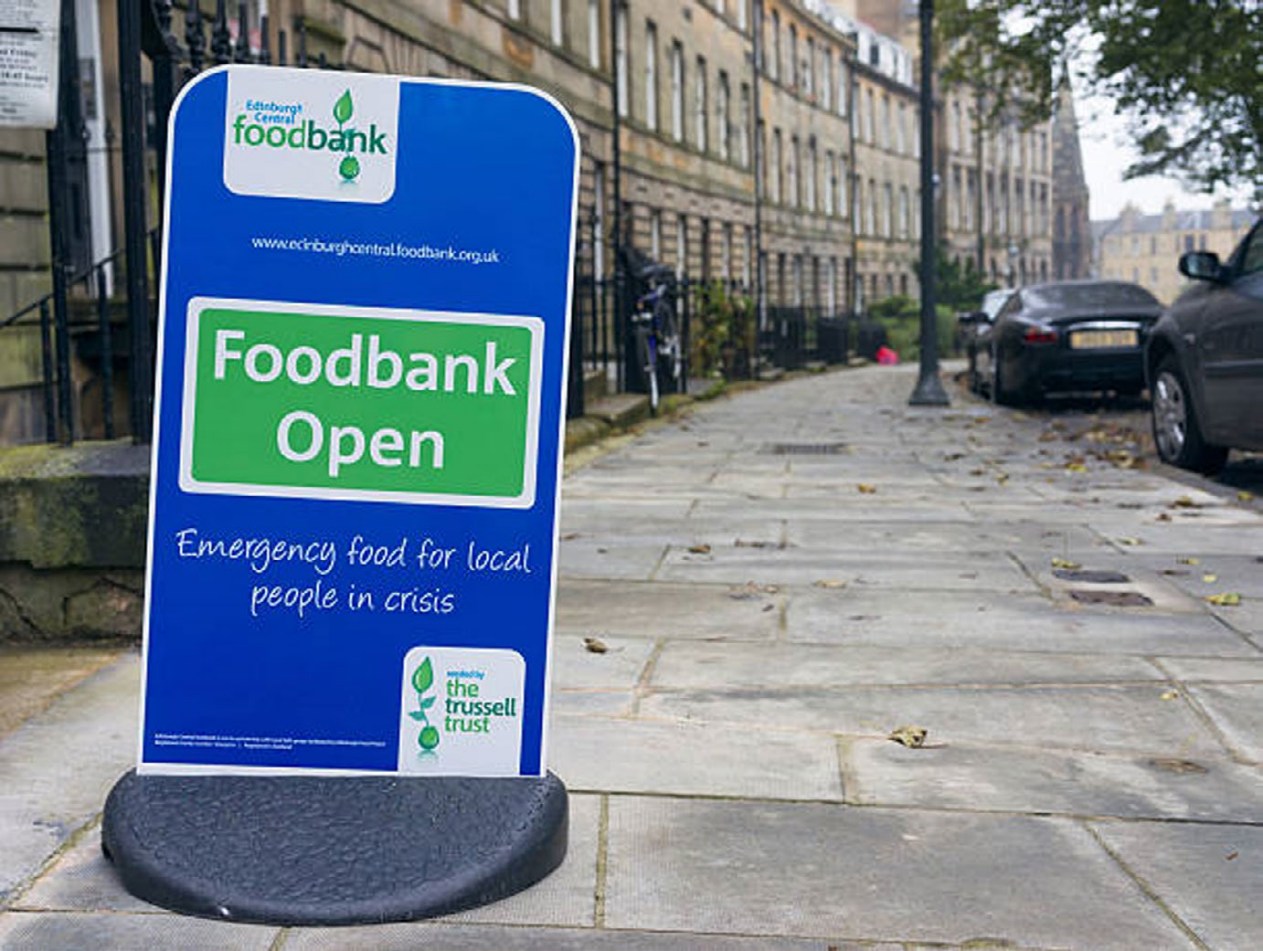
A prolonged recession is forecast in the wake of Covid‐19.
